# Association between joint hypermobility and primary nocturnal enuresis: a cross-sectional study in children aged 6–13 years

**DOI:** 10.1186/s12887-025-06175-6

**Published:** 2025-10-03

**Authors:** Dorna Derakhshan, Shabnam Hajiani Ghotbabadi, Fatemeh Mazarei, Ali Mirzakhanlouei, Faizan Bashir

**Affiliations:** 1 Department of Pediatric Nephrology, School of Medicine, Shiraz University of Medical Sciences, Shiraz, Iran; 2 Shiraz Nephro-Urology Research Centre, School of Medicine, Shiraz University of Medical Sciences, Shiraz, Iran; 3 Department of Pediatric Rheumatology, School of Medicine, Shiraz University of Medical Sciences, Shiraz, Iran; 4 Department of Pediatrics, School of Medicine, Shiraz University of Medical Sciences, Shiraz, Iran; 5 Department of Pediatric Urology, School of Medicine, Shiraz University of Medical Sciences, Shiraz, Iran; 6 School of Medicine, Shiraz University of Medical Sciences, Shiraz, Iran

**Keywords:** Nocturnal enuresis, Joint hypermobility, Beighton score, Urinary dysfunction, PNE

## Abstract

**Background:**

Nocturnal Enuresis (NE) is a prevalent childhood condition with a multifactorial pathogenesis comprising genetic, neurological, and connective tissue factors. Recent evidence points toward a possible link between joint hypermobility and NE, but the underlying mechanisms remain unclear, and existing data are limited. The objective of this study is to determine the prevalence of joint hypermobility in patients with primary nocturnal enuresis (PNE) relative to healthy controls and investigate potential correlations between these conditions.

**Methods:**

This cross-sectional study was conducted in 2024 at Imam Reza Clinic, the largest pediatric outpatient clinic affiliated with Shiraz University of Medical Sciences in Shiraz, Iran. A total of 180 children aged 6–13 years were recruited, including 90 children with primary nocturnal enuresis (study group) and 90 healthy children without nocturnal enuresis (control group). Participants were assessed for generalized joint hypermobility (GJH) using the Beighton score (≥ 6 indicating hypermobility). Demographic and clinical information was gathered on structured checklists. Statistical tests, such as chi-square tests, t-tests, and logistic regression, were carried out using SPSS (version 25) at a significance level of *p* < 0.05.

**Results:**

The prevalence of joint hypermobility was significantly higher in children with NE (87.8%) than in controls (28.9%) (*p* < 0.0001). Conversely, NE was present in 75.2% of hypermobile children compared with 14.7% of non-hypermobile children (*p* < 0.0001). Logistic regression analysis revealed that children with nocturnal enuresis were 19.87 times more likely to have joint hypermobility compared to non-enuretic children following the adjustment for age, gender, and BMI (*p* < 0.05). Gender-specific analysis indicated that hypermobile girls with nocturnal enuresis at a greater likelihood of suffering from urinary incontinence and frequent urinary tract infections (UTIs), whereas hypermobile boys with nocturnal enuresis had increased rates of constipation and urinary symptoms during the day.

**Conclusion:**

This study shows a strong association between GJH and PNE. Further research is needed to determine causal direction and underlying mechanisms.

## Introduction

According to the International Children’s Continence Society (ICCS), nocturnal enuresis is defined as intermittent incontinence that occurs during sleep at least once per month, for a minimum duration of three months, in children aged five years or older [1]. NE is divided into primary nocturnal enuresis (PNE), when children have never reached nighttime continence and secondary nocturnal enuresis (SNE), when enuresis recurs after at least six months of dryness [2–4]. It can further be categorized into monosymptomatic nocturnal enuresis (MNE), which occurs without additional lower urinary tract symptoms, and non-monosymptomatic nocturnal enuresis (NMNE), which is associated with symptoms such as daytime incontinence, urgency, or frequent urination [5, 6].

The prevalence of NE is depends on age, approximately 15% at age seven, 10% at age ten, 2% in adolescence, and about 0.5–1% in adulthood [5, 7, 8]. NE is more frequent in males, with a male-to-female ratio of 3:1; however, this difference decreases around age ten [5, 7, 8]. NE significantly affects children’s mental health, social development, general quality of life, and increases family stress [9].

NE is one of the most common urologic conditions affecting children, and its pathophysiology is considered complex and multifactorial. The primary mechanisms involved include nocturnal polyuria, impaired arousal, and reduced functional bladder capacity [10, 11].

Joint hypermobility is characterized by increased joint mobility beyond normal physiological range and is thought to be on a spectrum that includes localized, generalized (GJH), peripheral, and historical joint hypermobility [12–14]. GJH is the most clinically important type with well-defined diagnostic criteria. It is most commonly and broadly assessed using the Beighton scoring system [15]. The prevalence of GJH varies widely (2–57%) according to age, sex, and race [16], and about 10% of affected individuals have musculoskeletal or psychological manifestations [17]. In Iran, the prevalence of NE among children 3 to 18 years of age is approximately 10.2% [18]. Although historically viewed as a musculoskeletal disease, it is now increasingly recognized to impact numerous physiological systems, including the urinary system, gastrointestinal system, and autonomic nervous system [19].

Emerging hypotheses have suggested that structural factors, such as abnormalities in connective tissue like joint hypermobility, might affect bladder function and enuresis. A study conducted by de Kort et al. discovered that children with GJH had a significantly higher rate of lower urinary tract dysfunction (LUTD) compared to children without hypermobility [20]. Other pediatric case-control studies, including those by Pacey V and Kajbafzadeh AM, have also reported higher rates of urinary incontinence in children with joint hypermobility. However, a more recent study by Isik et al. (2024) did not find a statistically significant difference between the incidence of voiding dysfunction between children with or without joint hypermobility [21–23].

While both PNE and joint hypermobility are relatively common in children and might share underlying neurodevelopmental or connective tissue related mechanisms, their potential association remains poorly understood and under investigated. The current literature lacks robust data examining whether children with NE are more likely to exhibit GJH compared to their healthy peers.

The primary aim of this study was to assess the prevalence of joint hypermobility, as measured by the Beighton score, in children with primary NE and to compare it with age and sex matched controls. Additionally, the study also explored whether a statistically significant correlation existed between the presence of NE and GJH.

## Methods

This cross-sectional study primarily aimed to investigate the association between joint hypermobility and PNE in children aged 6–13 years by determining the prevalence of joint hypermobility in this population. Conducted in 2024 at Imam Reza Clinic, the largest pediatric outpatient center of Shiraz University of Medical Sciences (Shiraz, Iran), participants were recruited during their initial visit for NE evaluation and treatment.

### Participants and sampling

A total of 180 children were included in the study, divided equally into two groups: the study group (*n* = 90), comprising children diagnosed with primary nocturnal enuresis, and the control group (*n* = 90), consisting of children with no history of nocturnal enuresis. Children in the study group were recruited upon their first visit to Imam Reza Clinic for the confirmation and treatment of nocturnal enuresis, ensuring that they had not received prior treatment for the condition. The control group was recruited from the same clinic among children visiting for unrelated conditions, such as common cold, and were verified as enuresis-free through history-taking and clinical examination.

The control group was matched to the study group based on age and gender using the group matching method, with a ± 1 year range for age-matching.

### Inclusion and exclusion criteria

The inclusion criteria for the study group required children to have primary nocturnal enuresis, defined as involuntary urination during sleep in a child who has never achieved nighttime continence. The control group included children who, upon clinical examination, had no signs or history of nocturnal enuresis.

Children were excluded from the study if they met any of the following criteria:

*(1) age outside the study range (younger than 6 years or older than 13 years)*,* (2) unwillingness to participate*,* (3) presence of pre-existing conditions predisposing to joint hypermobility*,* including chronic constipation*,* multiple bone fractures*,* history of lens dislocation*,* Marfan syndrome*,* or Ehlers-Danlos syndrome*,* and (4) a diagnosis of UTI at the time of recruitment*.

### Control group screening

Control participants were recruited from the same pediatric outpatient clinic for non-urological concerns and were matched by age and sex. Medical histories were reviewed by pediatric nephrologists, with additional input from pediatric urologists or rheumatologists in cases where relevant comorbidities were suspected. Children with known or suspected urological, neurological, or musculoskeletal conditions were excluded based on physician assessment and caregiver report.

### Data collection and measurement tools

Data were collected using a structured checklist, which included demographic and clinical variables such as age, gender, height, weight, and presence or absence of NE. Joint hypermobility was assessed using the Beighton score, a standardized and validated tool for evaluating generalized joint laxity. The Beighton score consists of nine physical examinations, with one point awarded for each side where applicable (e.g., elbows, knees, thumbs, and fifth fingers), for a total of nine points. A total score of ≥ 6 considered indicative of joint hypermobility. The specific tests included:

1. *Hyperextension of the thumb to the forearm (bilateral)*
2. *Hyperextension of the fifth finger beyond 90° (bilateral)*
3. *Hyperextension of the elbows beyond 10° (bilateral)*
4. *Hyperextension of the knees beyond 10° (bilateral)*
5. *Ability to bend forward and touch the palms to the floor without bending the knees*

### Sample size calculation

The required sample size was determined based on the study by *Kajbafzadeh et al. (2014)*, considering a Type I error (α) of 0.05 and a power (1-β) of 99%, which resulted in a required sample size of 90 participants per group.

### Statistical analysis

All data were analyzed using SPSS software, version 25 (IBM Corp., Armonk, NY, USA). Descriptive statistics were used to summarize the data, with means (M) and standard deviations (SD) for continuous variables and frequencies (*n*) and percentages (%) for categorical variables. Inferential statistical tests were conducted as follows:

- ➢ *Chi-square test (χ² test)* was used to compare categorical variables, such as gender distribution and presence of joint hypermobility between groups.
- ➢ *Independent samples t-test* was applied to compare mean Beighton scores between the study and control groups.
- ➢ *Mann-Whitney U test* was used for non-parametric comparisons when data were not normally distributed.
- ➢*Multivariate logistic regression analysis* was used to assess the association between joint hypermobility and PNE while adjusting for potential confounders which included age, sex, and BMI.

There were no missing data for variables used in the analyses. A *p*-value < 0.05 was considered statistically significant for all analyses.

## Results

All analyses were carried out using SPSS version 25, with statistical significance set at *p* < 0.05.

### Demographic characteristics and hypermobility

**Fig. 1:**
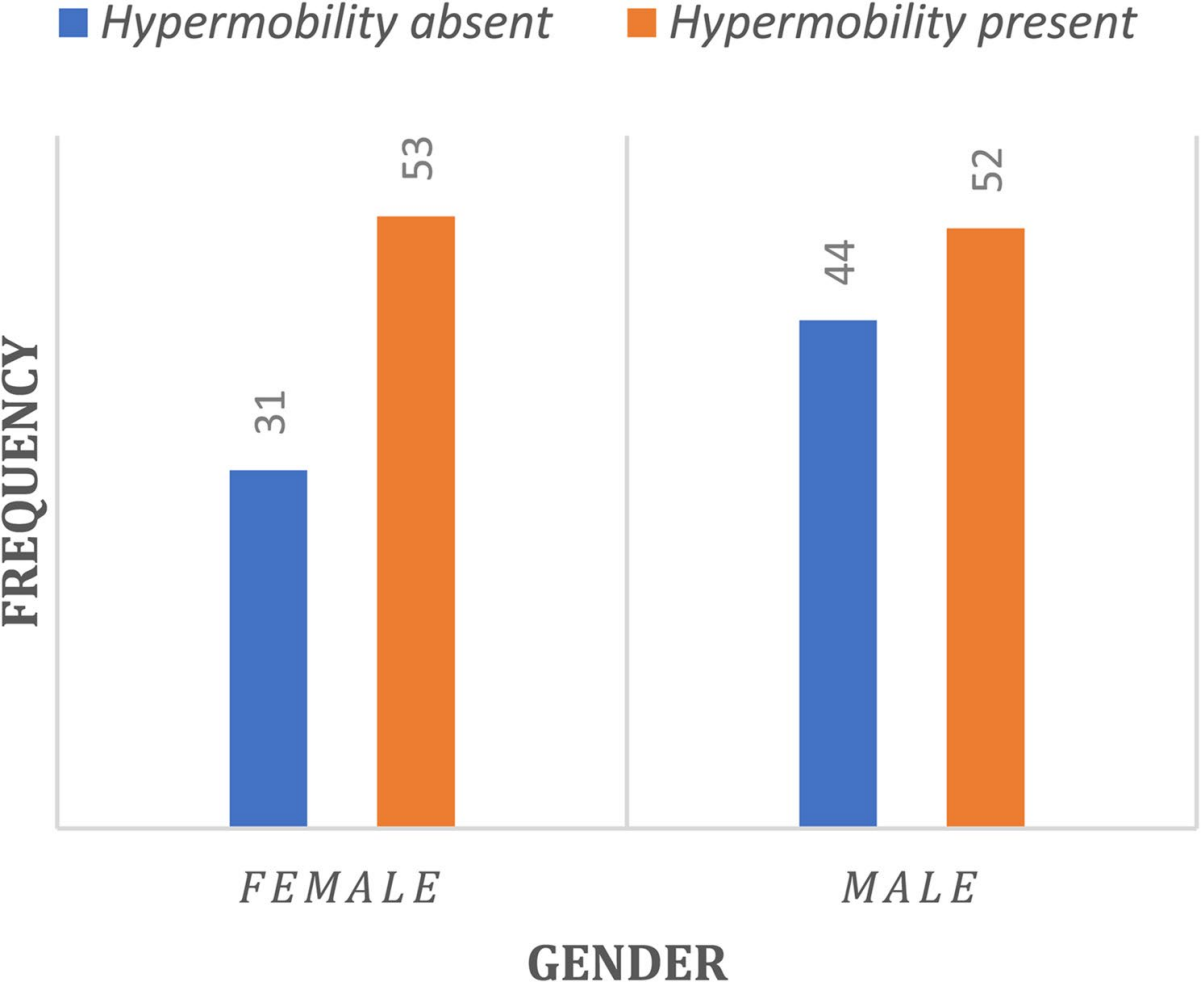
Frequency of Hypermobility by Gender. Bar chart comparing hypermobility frequencies between males and females. No statistically significant difference was observed (*p* = 0.225)

There were no significant associations between joint hypermobility and demographic variables, including age, sex, height, weight, or BMI (Table 1.) Both Chi-square and Mann–Whitney U tests yielded *p* > 0.05 across comparisons. As shown in Fig. 1, the prevalence of joint hypermobility was similar between males and females (*p* = 0.225), suggesting that sex does not significantly influence hypermobility status in this cohort.

**Table 1:** Comparison of demographic and clinical variables between children with and without joint hypermobility

Variable		Total population *n* = 180	Hypermobility		*P*-value
			Yes (*n = 105)*	No (*n = 75)*	
Gender, number (%)	*Female*	84 (46.7)	53 (50.5)	31 (41.3)	0.225*
	*Male*	96 (53.3)	52 (49.5)	44 (58.7)	
Nocturnal Enuresis, number (%)	*Absent*	90 (50)	26 (24.8)	64 (85.3)	< 0.0001*
	*Present*	90 (50)	79 (75.2)	11 (14.7)	
Age, Standard Deviation ± Average		8.3 ± 2.15	8.1 ± 2.07	8.57 ± 2.24	0.159**
Height, Standard Deviation ± Average		129.86 ± 13.69	128.58 ± 12.43	131.66 ± 15.19	0.274**
Weight, Standard Deviation ± Average		29.17 ± 11.89	27.18 ± 8.71	31.94 ± 14.91	0.205**
BMI, Standard Deviation ± Average		16.7 ± 3.91	16.12 ± 3.07	17.52 ± 4.75	0.282**

Values are presented as mean ± sd or number (%). *P*-values calculated using chi-square test for categorical variables and Mann–Whitney U test for continuous variables. BMI: body mass index

*: Chi-score test; **: Mann-Whitney Test

### Association between joint hypermobility and nocturnal enuresis

**Fig. 2:**
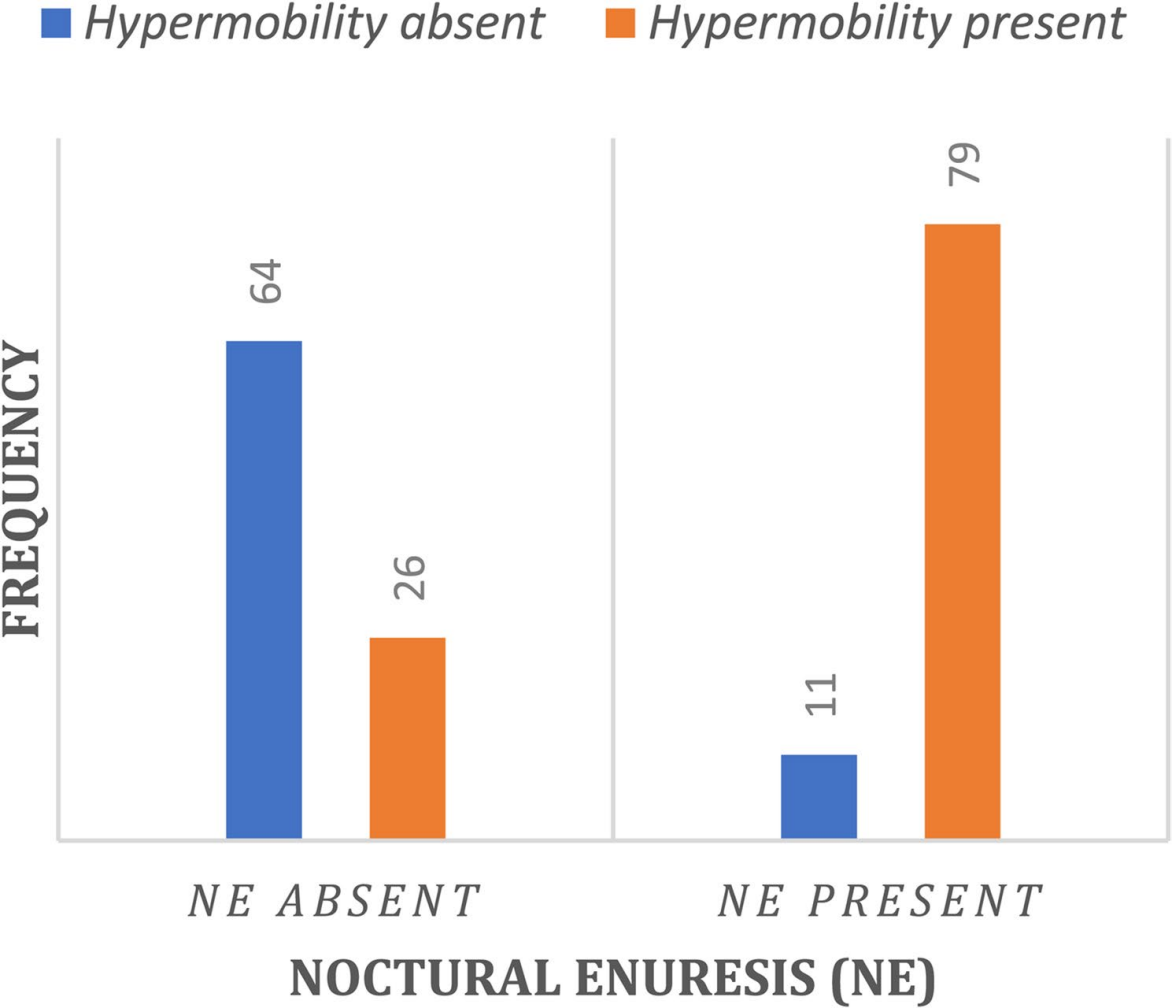
Frequency of Hypermobility by Enuresis. Bar chart illustrating the frequency of hypermobility among children with and without nocturnal enuresis. A significant association was observed (*p*< 0.0001)

In contrast, a strong and statistically significant association was observed between hypermobility and NE (Fig. 2). GJH was present in 79/90 (87.8%) of children with PNE and 26/90 (28.9%) of controls (crude OR = 17.7; 95% CI 8.12–38.50). Among hypermobile children, 75.2% had NE, compared to only 14.7% among non-hypermobile participants (*p* < 0.001), supporting the hypothesis that hypermobility is more frequently observed in children with enuresis.

### Stratified analyses by gender and age

Gender stratified analysis demonstrated a consistently higher prevalence of enuresis among hypermobile children in both sexes (Table 2.) Among hypermobile girls, 71.7% had enuresis versus 28.3% without, while in boys, the corresponding figures were 78.8% and 21.2%, respectively.

**Table 2:** Gender-stratified association between nocturnal enuresis and joint hypermobility

Variable				Hypermobility		*P*-value
				Yes *(n = 105)*	No *(n = 75)*	
Gender	Female	Enuresis	*Absent*	15 (28.3)	27 (87.1)	< 0.0001*
			*Present*	38 (71.7)	4 (12.9)	
	Male	Enuresis	*Absent*	11 (21.2)	37 (84.1)	< 0.0001*
			*Present*	41 (78.8)	7 (14.6)	

Values represent the frequency of enuresis among male and female participants with and without joint hypermobility. *P*-values calculated using chi-square test

When stratified by age group, the prevalence of enuresis among hypermobile children was 71.3% in those aged 6–9 years, and 88.0% in those aged 10–13 years, both significantly higher when compared with their non-hypermobile counterparts (28.7% and 12.0%, respectively) (Table 3.) These results indicate a higher prevalence of enuresis among hypermobile individuals, regardless of gender.

**Table 3:** Prevalence of nocturnal enuresis among children with and without joint hypermobility across different age groups

Variable				Hypermobility		*P*-value
				Yes *n* = 105	No *n* = 75	
Age	6–9 years	Enuresis	*Absent*	23 (28.7)	43 (82.7)	< 0.0001*
			*Present*	57 (71.3)	9 (17.3)	
	10–13 years	Enuresis	*Absent*	3 (12.0)	21 (91.3)	< 0.0001*
			*Present*	22 (88.8)	2 (8.7)	

Enuresis prevalence is shown for children with and without joint hypermobility, stratified by age group (6–9 and 10–13 years)

### Multivariate logistic regression

Logistic regression analysis showed that individuals with enuresis were 19.87 times more likely to have hypermobility compared to those without enuresis (OR = 19.87; 95% CI: 8.738, 44.941; *p* < 0.0001), as displayed in Table 4. In univariate analysis, BMI was inversely associated with GJH (OR = 0.91; 95% CI 0.84–0.99; *p* = 0.021); however, this association was not significant after adjustment for age and sex (OR = 0.90; 95% CI 0.80–1.01; *p* = 0.083). This suggests that BMI might not independently predict hypermobility when considering potential confounding factors.

**Table 4:** Univariate and multivariate logistic regression analysis of predictors of joint hypermobility

Variable		Univariate Logistic Regression			Multivariate Logistic Regression		
		OR	Confidence Interval 95%	*P*-value	OR	Confidence Interval 95%	*P*-value
Gender	*Female*	1.447	0.796–2.631	0.226	1.657	0.761–3.609	0.203
	*Male*	1	-		1	-	
Age	*6–9 years*	1.415	0.728–2.753	0.306	1.196	0.445–3.215	0.723
	*10–13 years*	1	-		1	-	
Enuresis	*Absent*	1	-	< 0.0001	1	-	< 0.0001
	*Present*	17.67	8.118–38.496		19.87	8.738–44.941	
BMI		0.91	0.841–0.986	0.021	0.9	0.799–1.014	0.083

Odds ratios (OR), 95% confidence intervals (CI), and *p*-values are shown for univariate and multivariate models assessing the association between joint hypermobility and gender, age, BMI, and enuresis

Neither age nor sex showed a significant association with joint hypermobility in the multivariate model. For example, female gender had an OR of 1.657 (95% CI: 0.761, 3.609; *p* = 0.203), and age 6–9 years had an OR of 1.196 (95% CI: 0.445, 3.215; *p* = 0.723).

### Summary of findings

When combined, these results demonstrate a robust and reliable correlation between children’s PNE and joint hypermobility. This correlation held true for both age and sex groups. In unadjusted analysis, BMI appeared to have a protective trend, but after adjustment, its impact was not statistically significant. The accompanying tables and figures provide a summary of all primary data.

.

## Discussion

The purpose of the present study was to determine the prevalence of GJH, as identified through the Beighton score, in patients with enuresis and a control group of healthy individuals. A significant association was found between joint hypermobility and NE (bedwetting). Specifically, 75.2% of children with joint hypermobility had NE, while only 24.8% of those without hypermobility experienced enuresis. The observed disparity was statistically significant. Multivariate logistic regression analysis identified NE as the only variable independently associated with joint hypermobility, with the odds of GJH being 19.87 times higher in children with NE compared to non-enuretic subjects (*p* < 0.05).

These findings indicate the presence of a significant relationship between GJH and NE. This connection is particularly noteworthy given that urinary dysfunction is prevalent in pediatric populations and influenced by behavioral, psychological, developmental, and genetic factors. Children with NE have a higher rate of joint hypermobility, suggesting urinary issues may stem from altered connective tissue properties similar to those seen in joint hypermobility. Particularly, pelvic floor dysfunction has been proposed as a likely mechanism of underlying urinary complaints, especially in women, although the underlying pathophysiology is uncertain [24, 25].

Joint hypermobility itself is usually a harmless condition but may on occasion be part of more severe disorders of connective tissue, including Ehlers-Danlos syndrome, Marfan syndrome, and osteogenesis imperfecta [26–28]. Understanding the connection between urinary dysfunction and joint hypermobility may help identify people who are at risk for both conditions and provide more targeted clinical care for them.

Even though our study primarily focused on NE, our results are consistent with those of Kajbafzadeh et al., who demonstrated that patients with urinary dysfunction had a substantially higher prevalence of GJH (45%) compared to healthy controls (17%) [23]. Their work supports the broader link between connective tissue laxity and urinary tract abnormalities. Additionally, their study found gender-specific trends in urinary dysfunction among GJH patients, with UTIs being more common in female patients and constipation being more prevalent in male patients with GJH.

Similarly, a study by de Kort et al. [20] identified that children with GJH had a greater incidence of sphincter dysfunction than a control group. In boys, this dysfunction was largely expressed as constipation and fecal incontinence, whereas in girls, urinary dysfunction typically presented as urinary incontinence and UTIs. Tokhmafshan et al. (2019) reported a higher prevalence of vesicoureteral reflux (VUR) among children with joint hypermobility, suggesting a potential overlap in underlying connective tissue vulnerabilities. Moreover, an Iranian study involving children with BJHMS reported that about 60% of participants also had VUR, further supporting the idea that connective tissue disorders and urinary tract abnormalities in children from this region may be related. More recently, Veriki et al. (2021) conducted a parent-reported survey in which enuresis emerged as a commonly observed symptom (41.7%) among children with symptomatic joint hypermobility. This study also highlighted the emotional burden of LUTD on affected children and their families [29–31].

The mechanism underlying joint hypermobility is thought to be due to defects in type I collagen synthesis. Type I collagen is the most abundant form in the human body and provides high tensile strength. In joint hypermobile patients, the proportion of type III collagen to type I collagen is elevated, which could be responsible for the laxity of connective tissues, such as those found in the pelvic floor [32]. Additionally, the genetic basis of hypermobility spectrum disorders (HSD) remain largely unknown and poorly understood [33].

Moreover, some earlier studies have shown an increased prevalence of genital-urinary prolapse, and urinary and fecal incontinence in females with joint hypermobility [34–36]. Ulmsten et al. [37] reported a 40% decrease in the collagen content of skin and round ligament biopsies in women with stress urinary incontinence compared to controls. Keane et al. [38] also showed a decrease in the content of collagen and the proportion of type I to type III collagen in vaginal biopsies from women with true stress urinary incontinence.

These results have key implications for clinical practice and future research. Given the strong association of joint hypermobility with urinary dysfunction, specifically NE, it is encouraged for clinicians to consider joint hypermobility in the assessment of pediatric patients with urinary symptoms. If joint hypermobility is identified early, it may assist in managing musculoskeletal complaints in addition to urinary complaints in this population.

Despite some limitations, including the lack of differentiation between monosymptomatic and non-monosymptomatic enuresis, there are other factors to consider. The cross-sectional design limits our ability to infer causal relationships. Although a thorough medical history was taken to exclude known urological or musculoskeletal conditions in controls, subclinical cases may not have been adequately recognized. Comorbidities like constipation and UTIs were assessed based on thorough clinical records and caregiver reports. Furthermore, we did not assess dietary variables that might influence the pathophysiology of enuresis, such as caffeine consumption or micronutrient levels. However, recent clinical trials, for example that of Rezakhaniha et al. [39] have suggested that limiting caffeine intake may reduce enuresis severity in children with PMNE, which warrants further investigation in studies with subtype stratification. Despite these limitations, we employed rigorous methods and controlled for major confounding factors.

Moreover, the link between joint hypermobility and pelvic floor dysfunction in girls warrants further investigation. Weakness in pelvic floor ligaments and soft tissues can lead to urethral inadequacy, which results in compensatory pelvic floor overactivity and urinary issues. These findings provide a strong basis for additional research to clarify the pathways from changes in connective tissues to urinary problems.

### Strengths & limitations

This study has notable strengths, including the use of a well-defined population, comprehensive statistical calculations and multivariate logistic regression to control for confounding variables. As mentioned earlier, a significant limitation of this study is the lack of stratification between monosymptomatic and non-monosymptomatic enuresis. These subtypes differ in pathophysiology, and joint hypermobility may play a more prominent role in non-monosymptomatic enuresis due to its association with pelvic floor dysfunction. Also, while this study did not assess voiding dysfunction, prior research indicates that joint hypermobility may be a contributing factor to LUTD, which may then mediate the observed association with NE. More research is required to fully understand this possible pathway. Future research should address this distinction to better understand the specific pathways involved.

## Conclusion

Generalized joint hypermobility was strongly associated with primary nocturnal enuresis in this matched, cross-sectional study. Longitudinal studies are warranted to determine temporality and underlying mechanisms.

## Data Availability

The data supporting the findings of this study are available upon reasonable request from the corresponding author, subject to ethical approval and confidentiality agreements.

## Abbreviations

BMI: Body mass index
GJH: Generalized joint hypermobility
JH: Joint hypermobility
LUTD: Lower urinary tract dysfunction
NE: Nocturnal enuresis
OR: Odds ratio
UTI: Urinary tract infection
BJHMS: Benign joint hypermobility syndrome
